# Comparative Assessment of the Efficacy of Commercial Hand Sanitizers Against Human Norovirus Evaluated by an *in vivo* Fingerpad Method

**DOI:** 10.3389/fmicb.2022.869087

**Published:** 2022-04-07

**Authors:** Blanca I. Escudero-Abarca, Rebecca M. Goulter, Clyde S. Manuel, Rachel A. Leslie, Kristen Green, James W. Arbogast, Lee-Ann Jaykus

**Affiliations:** ^1^Department of Food, Bioprocessing and Nutrition Sciences, North Carolina State University, Raleigh, NC, United States; ^2^GOJO Industries, Inc., Akron, OH, United States

**Keywords:** human norovirus, hand sanitizer, hand hygiene, fingerpad method, alcohol

## Abstract

Human noroviruses (hNoV) are the leading cause of acute non-bacterial gastroenteritis worldwide and contaminated hands play a significant role in the spread of disease. Some hand sanitizers claim to interrupt hNoV transmission, but their antiviral efficacy on human hands is poorly characterized. The purpose of this work was to characterize the efficacy of representative commercial hand sanitizers against hNoV using an *in vivo* fingerpad method (ASTM E1838-17). Eight products [seven ethanol-based and one benzalkonium chloride (BAK)-based], and a benchmark 60% ethanol solution, were each evaluated on 10 human volunteers using the epidemic GII.4 hNoV strain. Virus titers before and after treatment were evaluated by RT-qPCR preceded by RNase treatment; product efficacy was characterized by log_10_ reduction (LR) in hNoV genome equivalent copies after treatment. The benchmark treatment produced a 1.7 ± 0.5 LR, compared with Product A (containing 85% ethanol) which produced a 3.3 ± 0.3 LR and was the most efficacious (*p* < 0.05). Product B (containing 70% ethanol), while less efficacious than Product A (*p* < 0.05), performed better than the benchmark with a LR of 2.4 ± 0.4. Five of the other ethanol-based products (labeled ethanol concentration ranges of 62–80%) showed similar efficacy to the 60% ethanol benchmark with LR ranging from 1.3 to 2.0 (*p* > 0.05). Product H (0.1% BAK) was less effective than the benchmark with a LR of 0.3 ± 0.2 (*p* < 0.05). None of the products screened were able to completely eliminate hNoV (maximum assay resolution 5.0 LR). Product performance was variable and appears driven by overall formulation. There remains a need for more hand sanitizer formulations having greater activity against hNoV, a virus that is comparatively recalcitrant relative to other pathogens of concern in community, healthcare, and food preparation environments.

## Introduction

Human noroviruses (hNoV) are the leading cause of acute gastroenteritis worldwide (Pires et al., 2015), causing an estimated 684 million cases annually. The illness burden caused by hNoV represents a considerable cost to society, with recent estimates suggesting over $60 billion in societal impact globally, primarily in the form of loss of productivity due to illness (Bartsch et al., 2016). Because they can be transmitted readily *via* contaminated food, hNoV are also the leading cause of foodborne disease globally (Lopman et al., 2012; Pires et al., 2015), causing an estimated 5.5 million cases annually in the United States alone (Scallan et al., 2011). Immunity to hNoV is believed to be short-lived (Simmons et al., 2013) and while several vaccines are currently in development, none are yet widely available (Mattison et al., 2018). Thus, preventing transmission of hNoV relies on practices such as exclusion of sick individuals from workplaces and public settings, and adherence to sanitation and hygiene best practices.

A systematic review of outbreaks attributed to hNoV between January 2003 and July 2017 showed that food handlers contribute significantly to disease burden, with restaurants being the most common setting for hNoV outbreaks (Hardstaff et al., 2018). In particular, ready-to-eat foods, or those subjected to extensive human handling immediately preceding consumption, are common causes of outbreaks (Hall et al., 2014). In a recent study, it was reported that 53% of hNoV foodborne outbreaks are associated with poor personal hygiene of infected food handlers (Hall et al., 2012). In addition, enteric virus contamination of food could occur *via* the hands of pickers during manual harvesting such as, for instance, soft red fruits (Li et al., 2015) and green bell peppers (León-Félix et al., 2010). Collectively, it is clear that hands of infected food handlers are a major route of hNoV contamination of foods.

Washing hands with soap and water is universally accepted as an important hygiene measure for managing transmission of a variety of pathogens (Huang et al., 2014). In retail food settings in the U.S. (such as restaurants and grocery stores), handwashing with soap and water is considered the “gold standard” for performing hand hygiene. The U.S. Food and Drug Administration’s (FDA) Model Food Code (U.S. Food and Drug Administration, 2017), which serves as a regulatory framework for retail food handling across the country, provides strict guidelines related to handwashing for food handlers. Compliance with these guidelines in food handling environments remains extremely low. For example, a recent study found that 60–80% of foodservice establishments were out-of-compliance for employee handwashing (Verrill et al., 2021). Given the historical low compliance metrics with handwashing, and the fact that handwashing is the only permitted option for performing hand hygiene in retail food settings, there has been an interest in developing and evaluating alternative hand hygiene measures, including hand sanitizers (Allwood et al., 2016; Boyce and Schaffner, 2020). Taken together, there is a clear need for fast, convenient, and effective hand hygiene treatments for hNoV control on human hands in food retail, processing, and harvesting environments.

While not considered a replacement for proper handwashing for food handlers, hand sanitizers have long been recognized as an effective means of performing hand hygiene in many settings. The U.S. Centers for Disease Control and Prevention (CDC) recommends the use of hand sanitizers, especially those containing at least 60% alcohol, as an acceptable method of hand hygiene when soap and water are not readily available (U.S. Centers for Disease Control and Prevention, 2021). Hand sanitizers have several benefits relative to effective hand washing, including speed of use (Hilburn et al., 2003), convenience, skin mildness (Boyce, 2000; Boyce et al., 2000; Mukherjee et al., 2018) and broad-spectrum efficacy (for well-formulated products) (Macinga et al., 2008; Edmonds et al., 2012). Commercial hand sanitizers contain active ingredients intended to destroy or otherwise inactivate pathogens. The most common active ingredients are alcohol (either ethanol or isopropanol) and BAK (Macinga et al., 2008; Edmonds et al., 2012). While the active ingredient(s) is important to the efficacy of a hand sanitizer, overall product formulation is equally important, as illustrated by the fact that products with the same active ingredients have been shown to have vastly variable antimicrobial efficacy against a variety of viral and bacterial pathogens (Macinga et al., 2008).

While hand sanitizers may play an important role in controlling transmission of hNoV, their efficacies against this virus are poorly characterized *in vivo*. The purpose of this work was to characterize the efficacy of eight commercially available hand sanitizers, as well as a benchmark 60% ethanol solution, against GII.4 hNoV using an *in vivo* fingerpad method (ASTM E1838-17).

## Materials and Methods

### Hand Sanitizers

Eight commercially available hand sanitizers and a 60% ethanol solution (benchmark) were used in this study. The ethanol benchmark solution was prepared in the laboratory with neat ethanol and sterile filtered water to meet a final concentration 60.0% vol/vol. The test products’ active ingredients, inactive ingredients, and format (e.g., gel, foam, or liquid) are shown in Table 1. A foam dispenser was used when required for foam sanitizers.

**TABLE 1:** Commercial hand sanitizers evaluated in this study and their ingredients, format, and primary industry of application.

Product code	Product name	Manufacturer	Active ingredient as reported on product label	Inactive ingredients as reported on product label	Product pH as reported on product SDS and (pH as measured in the laboratory)	Product format
A	PURELL VF PLUS Hand Sanitizer Gel	GOJO Industries, Inc.	85% Ethanol (vol/vol)	Water Isopropanol Isopropyl myristate Caprylyl glycol Aminomethyl propanol Acrylates/C10-30 alkyl acrylate crosspolymer	8.8–10.3 (10.2)	Gel
B	PURELL VF481	GOJO Industries, Inc.	70% Ethanol (vol/vol)	Water Isopropyl alcohol Copper gluconate Diisopropyl sebacate PEG/PPg-20/6 dimethicone	3.8–5.2 (5.1)	Gel
C	PURELL Advanced Hand Sanitizer Gel	GOJO Industries, Inc.	70% Ethanol (vol/vol)	Water Isopropyl alcohol Capryl glycol Glycerin Isopropyl myristate, Tocophenyl acetate Acrylates/C10-30 alkyl acrylate crosspolymer Aminomethyl propanol	6.5–8.5 (8.3)	Gel
D	Germstar NORO	Soaptronic LLC	63% Ethanol (wt/wt)* *[68% Ethanol (vol/vol)])*	Water Isopropanol Emollient complex Fragrance	6.0–8.0 (6.7)	Liquid
E	Ecolab Quik-Care Foam Hand Sanitizer	Ecolab	62% Ethanol (wt/wt)* *[68% Ethanol (vol/vol)]*	Water PEG-10 dimethicone Ethyhexylglycerin farnesol Bisabolol, Tert-butyl alcohol Denatonium benzoate	6.0–9.0 (7.4)	Foam
F	Alcare Extra Foaming Sanitizer	Debmed	80% Ethanol (wt/wt)* *[85% Ethanol (vol/vol)]*	Water BIS-PEG-12 Dimethicone Citric acid Coco-glucoside Dihydroxypropyl PEG-5 linoleammonium chloride glyceril oleate Panthenol PEG-200 hydrogenated glyceryl palmate PEG-7 glyceryl cocoate	5.0–7.5 (7.6)	Foam
G	Ecolab Foam Hand Sanitizer	Ecolab	62% Ethanol (wt/wt)* *[68% Ethanol (vol/vol)]*	Water PEG-10 dimethicone Ethylhexylglycerin Farnesol Bisabolol Tert-butyl alcohol Denatonium benzoate	6.0–9.0 (8.3)	Foam
H	Ecolab Foodservice Foam Hand Sanitizer	Ecolab	0.1% Benzalkonium chloride (BAK)	Water Propylene glycol Isopropyl alcohol FD&C blue 1	5.0–9.0 (6.5)	Foam
Benchmark control	N/A	N/A	60% Ethanol (vol/vol)	Water	(7.5)	Liquid

*Ethanol concentration on the product label for these samples are reported as weight per weight (wt/wt). The ethanol concentrations for these products, expressed as volume per volume (vol/vol), are shown in italics and were calculated based on product density as measured in the authors’ laboratory.

### Human Norovirus Strain

The hNoV GII.4 Sydney strain, obtained as a deidentified stool specimen collected from a previous outbreak (courtesy of Dr. Shermalyn Greene, NC Department of Health and Human Services, Raleigh, NC) was suspended 20% in phosphate buffered saline (PBS) and used as inoculum directly on fingerpads.

### Fingerpad Assays

**FIGURE 1:**
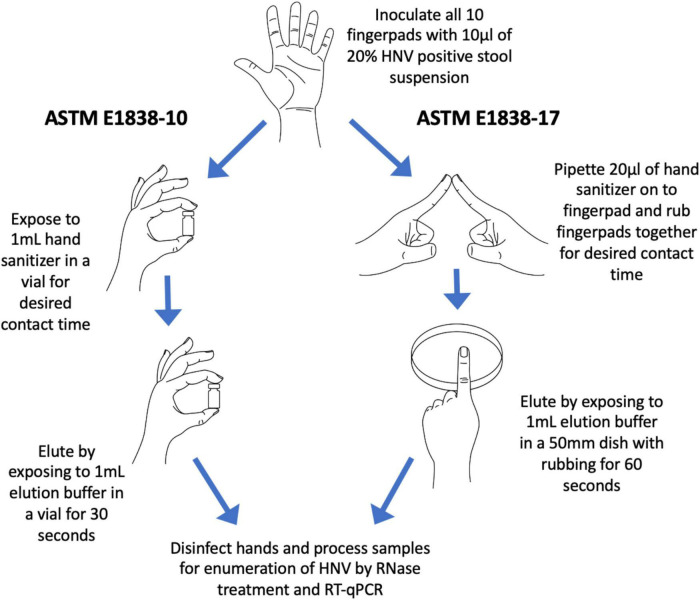
Diagram of the 2010 ASTM E1838-10 fingerpad method and the newer 2017 ASTM E1838-17 method. The two major differences between the methods are (1) ASTM E1838-10 does not include a rubbing step; and (2) virus elution is done by up-and-down inversions of buffer in a vial for ASTM E1838-10, and by rubbing fingerpads in a petri dish containing buffer for ASTM E1838-17.

Fingerpad assays were conducted in accordance with the ASTM International Standard E1838-17 (ASTM International, 2017; diagramed in Figure 1), with minor modifications for volume of inoculum and elution of virus from fingerpads. The study was approved by the North Carolina State University Institutional Review Board (IRB protocol number 16536) and informed consent was obtained from all participants, who were also compensated for participating in the study. Ten individual volunteers were recruited for each sanitizer tested, with a total of 21 individuals participating in the evaluation of the nine treatments included in this study. To be eligible to participate in the study, participants must have been 18–64 years of age; have short, clipped fingernails; have no known allergies to hand hygiene products; and not have used antimicrobial products on their hands for a period of 24 h before the study. The North Carolina State University IRB did not permit the collection of demographic data of participants. Only one product was tested on any one volunteer on any given day.

Briefly, decontamination of hands was done by washing with a non-medicated soap (Softsoap, Colgate-Palmolive, New York, NY) followed by placing 3–5 ml of 70% (vol/vol) ethanol in the palm of one hand and rubbing over the entire surface of both hands until the alcohol solution evaporated. An empty cryovial (2 ml volume, Thermo Fisher Scientific, Waltham, MA) was pressed onto each fingerpad, and the demarcated area drawn using an alcohol-proof marker. For the wet control, a 10 μl volume of hNoV inoculum was pipetted onto each thumb, then immediately eluted by gently rubbing the fingerpad on the bottom of a sterile 50 mm polystyrene petri dish (Cole-Parmer, Vernon Hills, IL) containing 1 ml of Earle’s Balanced Salt Solution with 0.1% Tween 20 (EBSS-T) for 60 s (wet inoculum control). The entire eluant volume was then transferred to a sterile 2 ml cryovial and placed on ice. After decontaminating the thumbs by pressing onto a folded paper towel soaked with 10% bleach for 3 min, each of the other fingerpads were inoculated with 10 μl of hNoV inoculum which was allowed to dry (approximately 30 min). The virus on the two index fingerpads was eluted immediately after drying to serve as the dry control. The other two middle fingers, which constituted “treatments” were exposed to the test product. Test products were applied to a treatment fingerpad by pipetting 20 μl of gel or liquid or by dispensing a 20 μl volume of foam product using a foam dispenser to the demarcated, inoculated area. The exposed fingerpad was then rubbed with an opposing inoculated fingerpad on the opposite hand, for a contact time of 30 s. The pinkies served as water rinse controls. After treatment, residual virus on each fingerpad was eluted as described above, by rubbing the fingerpads in a gentle back and forth motion on the bottom of petri dishes containing 1 ml EBSS-T for 60 s. Eluates were pre-treated with RNase to eliminate free RNA, providing a more accurate representation of presumptively infectious hNoV. For the RNase pre-treatment, 2 μl RNase One (Promega, Madison, WI) along with 22 μl of reaction buffer was added to 200 μl of the eluate and incubated at 37°C for 15 min. Samples were then placed on ice for 5 min to abolish RNase enzyme activity. RNase-treated samples were stored at −80°C until RNA extraction and enumeration were performed as described below. Fingerpads were decontaminated by pressing into paper towels soaked with 10% bleach for 3 min. A diagram of the ASTM 1838-17 method (ASTM International, 2017) and its comparison to the prior ASTM E1838-10 method (ASTM International, 2010) is shown in Figure 1.

### RNA Extraction and RT-qPCR

The automated EasyMag system (bioMerieux, Durham, NC) was used for RNA extraction as per manufacturer instructions, with a final RNA reconstitution volume of 25μl in NucliSENS^®^ elution buffer. Viral RNA was amplified by RT-qPCR targeting the conserved ORF1-ORF2 junction of hNoV GII as previously described (Jothikumar et al., 2005; Escudero-Abarca et al., 2020). For quantification, the resulting C_*T*_ values were extrapolated to log_10_ genome equivalent copies (GEC) by comparison to a standard curve produced by serial dilutions of hNoV GII.4 Sydney RNA obtained from the initial inoculum. Reduction in hNoV GEC as a function of treatment was calculated by subtracting the remaining virus log_10_ GEC for each treatment from that obtained for the dry control (baseline).

### Statistical Analysis

Results are presented as the mean ±standard deviation of log_10_ hNoV GEC reduction for each product (*n* = 10). These were compared statistically using ANOVA and the Tukey-Kramer test where the means of each treatment (including the 60% ethanol benchmark) were compared to the means of every other treatment (Minitab Statistical Software, State College, PA). Statistical significance was established at a level of *p* < 0.05.

## Results

**FIGURE 2:**
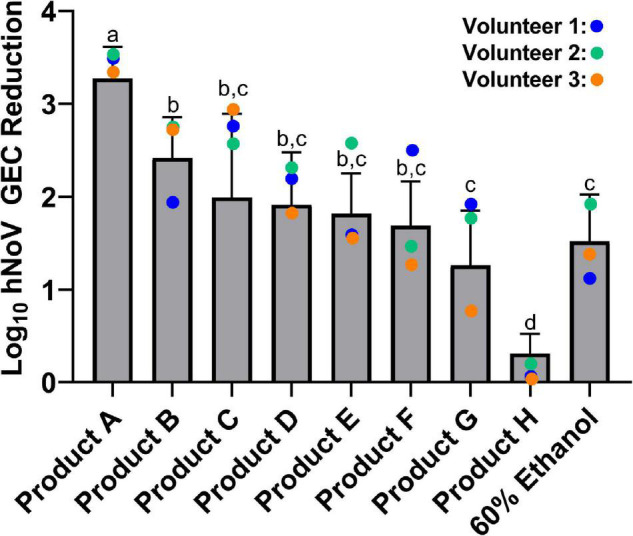
Efficacy of eight commercially available hand sanitizer products and a 60% ethanol benchmark solution. Results from a 30 s exposure are expressed as log_10_ hNoV GEC reduction ± standard deviation, as evaluated by the *in vivo* fingerpad assay ASTM E1838-17. Different letters indicate statistically significant differences in product efficacy (*p* < 0.05) by pairwise analysis. Colored dots refer to results for the three individual volunteers who participated in the evaluation of all products tested.

Product efficacies ranged from less than 0.5 log_10_ hNoV GEC reduction to 3.3 log_10_ hNoV GEC reduction (Figure 2). For the 30 s exposure time evaluated in this study, Product A [85% ethanol (vol/vol)], was the most efficacious (*p* < 0.05), with a log_10_ hNoV GEC reduction of 3.3 ± 0.3. Product B [70% ethanol (vol/vol)] was the only additional product found to be more efficacious than the 60% ethanol benchmark (*p* < 0.05) with a log_10_ hNoV GEC reduction of 2.4 ± 0.4. The performance of Products C through F, with log_10_ hNoV GEC reductions in the range of 1.7–2.0, differed significantly from Product A (*p* < 0.05) but not when compared to Product B or the 60% ethanol control (*p* > 0.05). The performance of Product G, with a log_10_ hNoV GEC reduction of 1.3 ± 0.6 was not significantly different from that of the 60% control (*p* > 0.05), but this product had a lower performance when compared to Products A and B (*p* < 0.05). Comparatively, Product H performed significantly worse than all other products included in the study, including the 60% ethanol control (*p* < 0.05), with a log_10_ hNoV GEC reduction of 0.3 ± 0.2.

Three individuals participated in the evaluation of all nine treatments included in this study. Results for Volunteers #2 and #3 largely followed the trends of the overall data (Figure 2). However, results for Volunteer #1 were often skewed from the general trends. For example, Product B produced a log_10_ hNoV GEC reduction of 2.0 on Volunteer #1 as compared to a mean log_10_ hNoV GEC reduction of 2.4 ± 0.4. Conversely, Product F produced a 2.5 log_10_ hNoV GEC reduction on Volunteer #1, with a mean log_10_ hNoV GEC reduction of 1.7 ± 0.5.

## Discussion

Hand hygiene is an important means by which to reduce transmission of hNoV, the leading cause of acute viral gastroenteritis and also foodborne disease (Scallan et al., 2011; Hall et al., 2013). While proper hand washing with soap and water is considered the gold standard hand hygiene intervention, it has been shown that compliance with handwashing requirements in certain food handling settings, such as restaurants, is low (Allwood et al., 2016; Boyce and Schaffner, 2020). Although hand sanitizers are not a substitute for handwashing by food handlers in the retail food sector, they may be used by food handlers after performing a handwash, which has been shown to significantly improve the overall efficacy of the hand hygiene treatment (Edmonds et al., 2016). Additionally, they may be used in the lobby, entrance, dining area, or restrooms (which can serve as a virus reservoir) of a retail food environment to help disrupt the environmental transmission of hNoV caused by infected patrons and guests.

The most common active ingredient in commercial hand sanitizers is ethanol, which has been shown to be very effective against bacteria (Davis et al., 2006) and enveloped viruses (Kampf, 2018). For example, a concentration of 42.6% ethanol (wt/wt) for a contact time of 30 s has been shown to be effective against SARS coronavirus-2, MERS coronavirus, and influenza A viruses (Kampf, 2018). On fingerpads, alcohol-based hand sanitizers (ABHSs) have been shown to be effective against bacteria including *Staphylococcus aureus*, *Pseudomonas aeruginosa*, *Escherichia coli*, *Serratia marcescens*, and *Acinetobacter baumannii* (Rotter, 1984; Ayliffe et al., 1988; Cardoso et al., 1999; Paulson et al., 1999). While ethanol as an active ingredient may be effective against certain bacteria and viruses at relatively low concentrations, it is worth mentioning that the U.S. FDA, which regulates the manufacture and sale of hand sanitizers as over-the-counter (OTC) drugs, requires ethanol-based hand sanitizers to be formulated with no less than 60% ethanol (vol/vol) (U.S. Food and Drug Administration, 2020).

Another common active ingredient in hand sanitizers are quaternary ammonium compounds (QACs). While QACs are effective against bacteria such as *S. aureus, P. aeruginosa*, and *E. coli*, among others (Ayliffe et al., 1988; Bondurant et al., 2020; Aodah et al., 2021), their efficacy against non-enveloped viruses such as poliovirus, murine norovirus, and hNoV appear be relatively poor (Feliciano et al., 2012; Tuladhar et al., 2012; Tung et al., 2013; Ha et al., 2016; Lee et al., 2021). The relative inefficacy of Product H (Figure 2), the only product in our study formulated with a QAC as an active ingredient, provides further support of the inability of this active ingredient to inactivate hNoV.

Evaluating sanitizer efficacy against hNoV is complicated by access to relevant virus strains and the general absence of an affordable and simple *in vitro* cultivation system. For these reasons, most previous studies characterizing the anti-hNoV efficacy of hand sanitizers include only *in vitro* suspension assays, or *in vivo* fingerpad studies using cultivable surrogates such as feline calicivirus (FCV), murine norovirus (MNV) or bacteriophages (MS2) (Macinga et al., 2008). Many of these surrogates behave differently as compared to hNoV when exposed to different chemicals or product formulations. For example, FCV is a respiratory pathogen and is less acid tolerant than is hNoV; MNV is more sensitive to alcohol than is hNoV; and bacteriophage MS2 is highly resistant to ethanol (Cromeans et al., 2014). Those few studies done with hNoV show a general trend of poor efficacy for both alcohol-based products (Liu et al., 2009, 2011; Tuladhar et al., 2015) as well as QACs (Girard et al., 2010; Tung et al., 2013). However, with careful formulation, a few recent alcohol-based products have shown comparatively better efficacy against hNoV compared to earlier formulations (Liu et al., 2011; Escudero-Abarca et al., 2020).

The purpose of this study was to compare the efficacy of seven commercial alcohol-based hand sanitizers, with varying concentrations of ethanol (labeled in the range of 62–85%), one commercial hand sanitizer containing the QAC BAK (0.1% BAK), and a 60% ethanol solution used as a benchmark control, on human fingerpads against hNoV using the *in vivo* assay ASTM E1838-17. Previous studies have utilized earlier versions of the ASTM E1838 fingerpad method (ASTM E1838-10 or earlier) to evaluate the efficacy of hand sanitizers (Macinga et al., 2008; Liu et al., 2009, 2011). The major differences between the two protocols are (1) the addition of friction (rubbing) during the sanitizer application step for the newer protocol; and (2) the use of an alternative elution method (Petri dish vs. cryovial) (Figure 1). The justification for relevance of the newer (2017) method is that the addition of the rubbing step is more representative of sanitizer use in real world settings and increases the degree of exposure of the product to the inoculum.

The inactivation of hNoV in suspension by simple ethanol solutions has been studied extensively, with concentrations up to 90% (vol/vol) failing to show significant reductions in hNoV, usually less than 0.5 log_10_ hNoV GEC (Tung et al., 2013), although there are strain-to-strain differences in product efficacy (Park et al., 2016). In our study, all alcohol-based hand sanitizers demonstrated over 0.5 log_10_ hNoV GEC reduction, most approaching 2 log_10_ hNoV GEC reduction. In this case, alcohol content alone did not necessarily dictate efficacy against hNoV. Converting wt/wt to vol/vol for comparative purposes (Table 1), for example, Product F, which had an ethanol content of 85% (vol/vol), showed a 1.7 ± 0.5 log_10_ hNoV GEC reduction. This was statistically less of a reduction as compared to Product A, despite both products having similar ethanol content. Additionally, the efficacy of Product F was not statistically different than Products B, C, D, E, and G, despite having a higher ethanol content [85% (vol/vol) vs. 68–70% (vol/vol), respectively], These data are illustrative of the concept that hand sanitizer efficacy is a function of both active ingredient concentration and product formulation. Indeed, many factors related to product formulation (e.g., inactive ingredients such as skin conditioners and thickeners) impact the overall efficacy of the final product (Macinga et al., 2008; Edmonds et al., 2012). Various compounds, including mixtures of alcohols, metals such as copper and silver, or citric acid, may produce increases in the antiviral activity of ethanol, or act with ethanol in a complimentary or synergistic manner (Macinga et al., 2008; Escudero-Abarca et al., 2020).

Product A statistically outperformed all other products tested (*p* < 0.05), demonstrating a greater than 3 log_10_ hNoV GEC reduction. While the exact mechanism is unknown, it is likely (as mentioned above) due to the total product formulation boosting the effectiveness of the ethanol as the active ingredient. Product A was the only product specifically designed with an alkaline pH (8.8–10.3). Product B was specifically designed with an acidic pH (3.8–5.2) and the remaining products included in this study fell in a more neutral pH range (5.0–9.0) (Table 1). It has been previously shown that alcohol-based hand sanitizer efficacy against hNoV can be enhanced by adjusting the product’s pH to either acidic or alkaline conditions (Sato et al., 2020), presumably due to the pH extremes altering the capsid morphology in a way that exposes amino acid residues, allowing them to be more vulnerable to active ingredients than at pH neutral conditions. Even with relatively high efficacy, this product did not completely eliminate detectable hNoV from the fingerpads of volunteers (maximum assay resolution 5.0 log_10_ hNoV GEC reduction). Whether this is an artifact of using RNase-RT-qPCR as the virus quantification method, or is truly associated with incomplete virus inactivation, remains unknown. While it would have been interesting to additionally evaluate a “low level” of initial contamination of fingerpads to determine if inactivation trends remained similar to those observed for the “high level” of fingerpad contamination evaluated in this study, the loss of assay resolution using a lower starting inoculum would have made evaluating these treatments much more difficult, and made it more challenging to measure statistically significant differences. While a successful hNoV cell culture model has been described recently (Ettayebi et al., 2016; Costantini et al., 2018), its routine use is limited by high cost, complexity, sensitivity to cytotoxicity, a limited number of cultivable strains, and the inability to produce quantitative results in the form of log_10_ reduction in infectious virus. Nonetheless, this model was recently used to validate the efficacy of Product A against hNoV (Escudero-Abarca et al., 2020), providing data complementary to that produced by RNase-RT-qPCR. In that study, hNoV infectivity was abolished in the cell culture model following exposure to Product A in suspension for 60 s. When evaluated by RNase-RT-qPCR, the same study demonstrated a log_10_ reduction in hNoV GEC of 2.3–3.2 when exposed to Product A (with and without additional soil load) for 30–60 s in suspension. This was compared to the 0.6–0.9 log_10_ reduction in hNoV GEC following exposure to the 60% ethanol benchmark under the same treatment conditions (Escudero-Abarca et al., 2020). Interestingly, the 3.3 log_10_ reduction of hNoV on fingerpads by Product A in the current study is almost identical to the 3.2 log_10_ reduction observed in the previous study for a 30 s exposure in suspension without additional soil (Escudero-Abarca et al., 2020), demonstrating that the product seemingly does retains efficacy on human fingerpads.

A unique aspect of this study was that three of the ten volunteers participated in the evaluation of all nine hand sanitizer treatments, making it possible to compare efficacy of all the products amongst these three volunteers (Figure 2). For some products, log_10_ hNoV GEC reduction was quite similar between the volunteers, for others, there was up to a 2 log_10_ difference in calculated hNoV GEC reduction between volunteers. In addition, the overall trends in product efficacy largely stayed consistent for volunteers #2 and #3, however, for volunteer #1, results seemed to skew from the trends observed for the group as a whole. In other words, the products did not consistently perform better on one volunteer over another, suggesting that differences in skin properties such as skin hydration level, skin pH and/or skin microbiome (Mukherjee et al., 2018) may influence the efficacy of different product formulations. Similar to data observed using *in vivo* fingerpad methods and bacteria, our results support the fact that subjects are a large source of variability when the same methods are applied to evaluating anti-hNoV activity *in vivo* (Rotter, 1984; Suchomel et al., 2018). In order to control for this effect, studies should be appropriately sized and, ideally, be cross-over designs where all subjects are evaluated with all treatments, and their individual results can be compared. However, it is also recognized that these criteria may be difficult to meet using a population of human volunteers.

## Conclusion

In conclusion, the results of this study support the long-held belief that not all hand sanitizers have anti-hNoV efficacy, and those that do may not completely eliminate the virus from fingerpads. It is not understood how the reductions of hNoV by hand sanitizer on fingerpads compares to reductions by handwashing, the current recommended practice. Further studies investigating the individual and combined effects of handwashing and hand sanitizer use on the removal and inactivation of hNoV on human hands are warranted, particularly studies that capitalize on hNoV cultivation as new culture methods are refined. There remains a need for more hand sanitizer formulations having greater activity against hNoV, a virus that is comparatively recalcitrant relative to other pathogens of concern in community, healthcare, and food preparation environments.

## Data Availability Statement

The raw data supporting the conclusions of this article will be made available by the authors, without undue reservation.

## Ethics Statement

The studies involving human participants were reviewed and approved by the North Carolina State University Institutional Review Board. The patients/participants provided their written informed consent to participate in this study.

## Author Contributions

BE-A performed laboratory-based experiments and data collection. RG, L-AJ, RL, and CM performed data analysis. BE-A prepared the first draft of the manuscript, while RG, L-AJ, RL, CM, KG, and JA reviewed and revised prior to submission. All authors contributed to project conception and design of experiments. All authors contributed to the article and approved the submitted version.

## Conflict of Interest

RL, CM, KG, and JA were full time employees of GOJO Industries as scientists. The remaining authors declare that the research was conducted in the absence of any commercial or financial relationships that could be construed as a potential conflict of interest.

## Publisher’s Note

All claims expressed in this article are solely those of the authors and do not necessarily represent those of their affiliated organizations, or those of the publisher, the editors and the reviewers. Any product that may be evaluated in this article, or claim that may be made by its manufacturer, is not guaranteed or endorsed by the publisher.
